# Mitochondrial Delivery of Phenol Substructure Triggers Mitochondrial Depolarization and Apoptosis of Cancer Cells

**DOI:** 10.3389/fphar.2018.00580

**Published:** 2018-06-04

**Authors:** Elena Gazzano, Loretta Lazzarato, Barbara Rolando, Joanna Kopecka, Stefano Guglielmo, Costanzo Costamagna, Konstantin Chegaev, Chiara Riganti

**Affiliations:** ^1^Department of Oncology, University of Turin, Turin, Italy; ^2^Department of Drug Science and Technology, University of Turin, Turin, Italy

**Keywords:** triphenylphosphonium cation, phenols, cancer mitochondrial biogenesis, cancer mitochondrial energy metabolism, mitochondrial-dependent apoptosis

## Abstract

Antitumor chemotherapy remains one of the most important challenge of the medicinal chemistry. Emerging research in chemotherapy is focused on exploiting the biochemical differences between cancer cell and normal cell metabolism in order to reduce the side effects and increase antitumor therapy efficacy. The higher mitochondrial transmembrane potential of cancer cells compared to not-transformed cells favors the intra-mitochondrial accumulation of cationic drugs in the former. This feature could be exploited to allow selective delivery of antineoplastic drugs to the cancer cells. In this work we designed and synthetized phenol derivatives joined to the triphenylphosphonium (TPP) cation, a well-known vector for mitochondrial targeting. Two designed phenol TPP-derivatives **1** and **2** show remarkable cytotoxic activity against different cancer cell lines, but were less toxic against normal cells. The differential cytotoxicity relied on the higher mitochondrial biogenesis and oxidative-phosphorylation metabolism of the former. By reducing mitochondrial mass and energetic metabolism, and increasing at the same time the levels of intra-mitochondrial reactive oxygen species, phenol TPP-derivatives **1** and **2** induced mitochondria depolarization and triggered a caspase 9/3-mediated apoptosis, limited to cancer cells. This work provides the rationale to further develop phenol TPP-derivatives targeting mitochondria as new and selective anticancer tools.

## Introduction

The vast majority of chemotherapy drugs targets macromolecules important for cell survival and proliferation, such as DNA-replication machinery and cytoskeleton structures. While this approach effectively kills cancer cells, it also affects rapidly growing normal cells producing several toxic side effects. Emerging research in chemotherapy is focused on exploiting the biochemical differences between cancer cell and normal cell metabolism in order to increase the ratio between anti-tumor benefits and undesirable side effects (Hsu and Sabatini, 2008).

A key metabolic change occurring during neoplastic transformation is the increased energetic demand, paralleled by the shift from oxidative phosphorylation to aerobic glycolysis (the Warburg effect) (Warburg, 1956). Despite it has been generally accepted that most solid tumors obtain fuel from aerobic glycolysis, the role of mitochondrial oxidative metabolism has been recently re-evaluated as a key contributor in tumor growth and progression (Trotta and Chipuk, 2017). For instance, a functionally active oxidative mitochondrial metabolism promotes tumorigenesis, cell migration, metastasis (Caino et al., 2015; García-Ledo et al., 2017), and drug resistance (Riganti et al., 2015a; Buondonno et al., 2016). By contrast, impairing mitochondria energetic metabolism and ATP synthesis, may reduce cancer cell proliferation and invasion, and trigger apoptosis (Trotta and Chipuk, 2017). For this reason, targeting tumor cells mitochondria and impairing mitochondria energetic metabolism became one of the most promising strategy in the antitumor therapy (Wallace, 2012; Riganti et al., 2015a; Weinberg and Chandel, 2015; Buondonno et al., 2016; Refaat et al., 2017).

Metabolically active mitochondria have highly negative membrane potential. Charged molecules are generally unable to cross cell membranes without the aid of transporter proteins, due to the large activation energies associated with removal of associated water molecules. The delocalization of charge across the large lipophilic surface of organic cations significantly lowers this energy requirement, facilitating passage across lipid membranes (Murphy, 2008). Thus, TPP cation, which easily passes through cellular membrane and accumulates in mitochondria, has been used as a targeting vector for the mitochondrial drug delivery (Rideout et al., 1989; Bergeron et al., 2009). Enhanced accumulation of cationic drugs in the solid tumors, when compared to their normal counterparts, has been attributed to the higher (i.e., more negative inside) mitochondrial transmembrane potential of cancer cells compared to normal cells (Modica-Napolitano and Aprille, 2001). This feature could be exploited to allow selective delivery of antineoplastic drugs to the tumor cell.

The antitumor activity of TPP salts was reported for the first time in 1978 during the routine screening of some synthetic intermediates (Dubois et al., 1978). More recently, some TPP salts were reported to show anti-proliferative activity against cancer cell lines (Rideout et al., 1989; Manetta et al., 1996). These studies indicated that TPP salts could selectively accumulate in cancer cells and TPP itself is not cytotoxic. The recent study of different TPP salts, which screened 40000 candidates, has individuated three molecules endorsed with antitumor activity in the low micromolar range (Millard et al., 2010). Authors have synthetized a number of structural analogs of three molecules, but coherent structure activity relationship between them was not identified: the only cytotoxic mechanisms proposed was the inhibition of oxygen consumption, leading to increased free radical production and consequent apoptosis (Millard et al., 2010).

Taking into account the lack of cytotoxicity of TPP cation and the absence of clear structural requirements for the anti-proliferative activity of TPP salt, in this paper we conceived TPP-derivatives containing protected phenol functionality. Phenols normally act as antioxidants by scavenging free radicals with the formation of stable phenolic radical, but they could also become pro-oxidants and cytotoxic (Prochazkova et al., 2011). Some phenol derivatives structurally related to chromanol (the phenolic part of tocopherol) exhibit cancer preventive, anti-proliferative, and pro-apoptotic antitumor activity in xenograft tumor (Kunnumakkara et al., 2010; Ju et al., 2010; Li et al., 2011). The exact mechanisms of action of these derivatives remain unknown; however, various models have been put forth, ranging from their antioxidant and anti-inflammatory effects to altered redox-signaling (Azzi et al., 2002).

**FIGURE 1:**
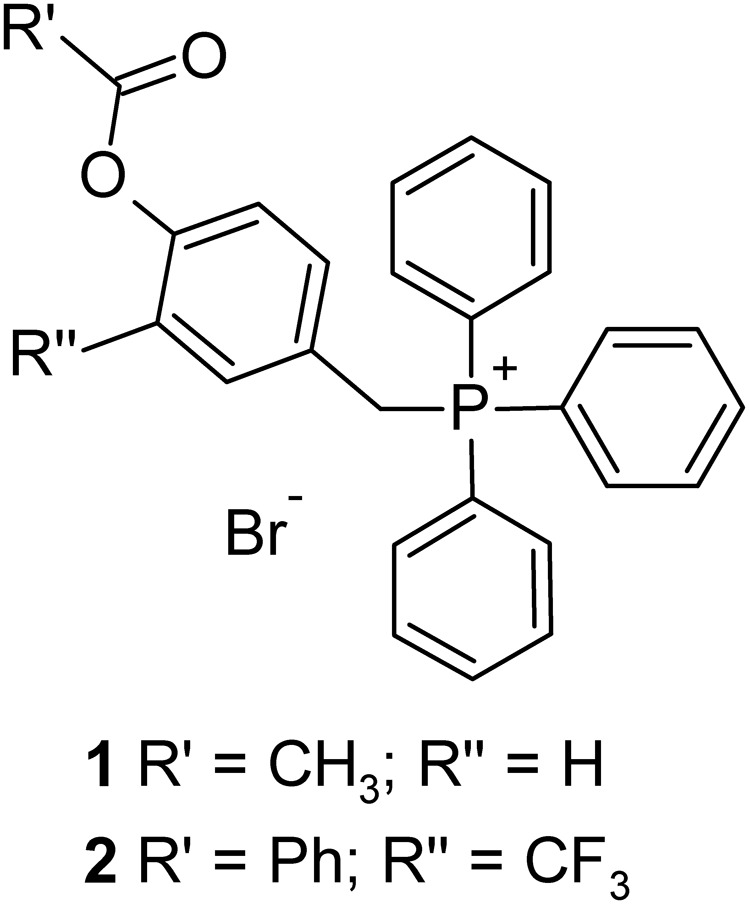
Phenolic esters TPP-derivatives.

On these bases, we designed and synthetized two phenol TPP-derivatives (**Figure 1**) where hydroxyl groups are protected as esters, able to accumulate in mitochondria and release the corresponding phenols by enzymatic hydrolysis. We investigated the cytotoxic activity of these compounds against different cancer cell lines and normal cells, and the biochemical mechanisms of their selective anti-cancer activity.

## Materials and Methods

### Chemicals

Fetal bovine serum and culture medium were from Invitrogen Life Technologies (Carlsbad, CA, United States). Plasticware for cell cultures was from Falcon (Becton Dickinson, Franklin Lakes, NJ, United States). The protein content in cell monolayers and mitochondrial extracts was assessed with the BCA kit from Sigma Chemical Co. (St. Louis, MO, United States). TPMP was purchased from TCI Europe N.V. (Zwijndrech, Belgium). Unless otherwise specified, all the other reagents were purchased from Sigma Chemical Co.

### Lipophilicity Measurements

The partition coefficient between *n*-octanol and PBS at pH 7.4 (log P) was obtained by shake-flask technique at room temperature. In the shake flask experiments 50 mM PBS pH 7.4 was used as aqueous phase; the organic (*n*-octanol) and aqueous phase (PBS) were mutually saturated by shaking for 4 h. The compounds were solubilised in the buffered aqueous phase at a concentration of 30 μg/ml and appropriate amounts of *n*-octanol were added. The two phases were shaken for about 20 min, by which time the partitioning equilibrium of solutes is reached, and then centrifuged (10000 rpm, 10 min). The concentration of the solutes was measured in the aqueous phase by UV spectrophotometer (UV-2501PC, Shimadzu); the values of absorbance (223, 268 and 275 nm) were interpolated in calibration curves obtained using standard solutions of compounds (concentration range 30–0.5 μg/ml; *r***^2^** > 0.99). Each log *P*-value is an average of at least six measurement.

### Cells

Human colon cancer HT29 cells, colon-derived normal fibroblast CCD-18Co cells, lung cancer A549 cells, normal human bronchial epithelial (immortalized with adenovirus 12-SV40 virus hybrid Ad12SV40) BEAS-2B cells, breast cancer MDA-MB-231 cells, normal breast epithelial MCF10A cells, human osteosarcoma U-2OS cells were purchased from ATCC (Manassas, VA, United States). Primary osteoblasts were generated as reported previously (Buondonno et al., 2016). HMM and HMC were primary cells stabilized in culture as described (Aldieri et al., 2004). The use of primary cells was approved by Ethical Committee of the San Luigi Gonzaga Hospital, Orbassano, (protocol #126/2016). All subjects gave written informed consent in accordance with the Declaration of Helsinki. Cells were maintained in medium supplemented with 10% v/v fetal bovine serum, 1% v/v penicillin-streptomycin, 1% v/v L-glutamine. Morphological analysis was performed with a bright field microscope (Leica Microsystems, Wetzlar, Germany). At least 5 fields/experimental condition were examined.

### Mitochondria Isolation

Mitochondria were isolated as reported (Riganti et al., 2013a). To isolate mitochondrial fractions, 5 × 10^6^ cells were washed twice in ice-cold PBS, then lysed in 0.5 ml buffer A (50 mM Tris, 100 mM KCl, 5 mM MgCl_2_, 1.8 mM ATP, 1 mM EDTA; pH 7.2), supplemented with protease inhibitor cocktail III (Calbiochem. La Jolla, CA, United States), 1 mM phenylmethylsulfonyl fluoride and 250 mM NaF. Samples were clarified by centrifuging at 650 × *g* for 3 min at 4°C. The supernatant was collected and centrifuged at 13,000 × *g* for 5 min at 4°C. This supernatant, containing the cytosolic fraction, was stored at -80°C until the use. The pellet containing mitochondria was washed in 0.5 ml buffer A and re-suspended in 0.25 ml buffer B (250 mM sucrose, 15 mM K_2_HPO_4_, 2 mM MgCl_2_, 0.5 mM EDTA, 5% w/v, BSA). A 50 μl aliquot was sonicated and used for the measurement of protein content or Western blotting; the remaining part was stored at -80°C until the use. To confirm the presence of mitochondrial proteins in the extracts and the absence of cytosolic contamination in the mitochondrial fraction, 10 μg of each sonicated sample were subjected to SDS–PAGE and probed with an anti-porin antibody (Abcam, Cambridge, United Kingdom), a mitochondrial marker, and with an anti-glyceraldehyde 3-phosphate dehydrogenase antibody (GAPDH; Santa Cruz Biotechnology Inc., Santa Cruz, CA, United States), a cytosolic marker. Mitochondrial extracts were used only if they had detectable levels of porin and undetectable levels of GAPDH. To exclude any mitochondrial contamination in the cytosolic extracts, the absence of porin and the presence of GAPDH in the latter was analyzed by Western blotting (**Supplementary Figure S1**). Nuclear proteins were extracted using the Nuclear Extract Kit (Active Motif, La Hulpe, Belgium). 10 μg of nuclear extracts were subjected to SDS–PAGE and probed with antibodies against: proliferator-activated receptor gamma coactivator 1-α (PGC-1α; Abcam), an index of increased mitochondrial biogenesis (Buondonno et al., 2016), or TATA box Binding Protein (TBP; Santa Cruz Biotechnology Inc.), as control of equal protein loading.

### Mitochondrial/Cytosolic Distribution

The amount of **1, 2, 15**, and **16** in the mitochondrial and cytosolic fractions was determined by LC-ESI-MS analyses. LC-ESI-MS analyses were performed with an Acquity Ultra Performance LC^TM^ (Waters Corporation, Milford, MA, United States), equipped with BSM, SM, CM, and PDA detector. All the chromatographic separations were performed on a Zorbax Eclipse XDB-C18 (5 μm, 150 mm × 4.6 mm) (Agilent Technologies) as a stationary phase. The supernatant samples obtained from incubation were filtered through a 0.45 μm pore size PTFE membrane filter before use. Aliquots (5 μl) were injected onto the system and eluted with a mobile phase (flow rate, 0.5 ml/min) consisting of A, 0.1% formic acid solution, and B, acetonitrile. The following gradient was used: 0–5 min (*A* = 50%, *B* = 50%), 5–7 min (to *A* = 20%, *B* = 80%), 7–8 min (*A* = 20%, *B* = 80%), 8–10 min (to *A* = 50%, *B* = 50%). The eluate was injected into the electrospray ion source (ESI), and monitored using Micromass Quattro microTM API ESCi multi-mode ionization Enabled as detector. MS spectra were acquired and processed using MassLynx software. The operating conditions on the triple quadruple mass spectrometer were as follows: positive mode; drying gas (nitrogen) heated at 350°C at a flow rate of 800 l/h; nebulizer gas (nitrogen) at 80 l/h; capillary voltage in positive mode at 3000 V; cone voltage at 30 V. The molecular ion [M]**^+^** was employed for quantitative measurements of analytes. The values obtained from integration of the peak of compounds were interpolated in a calibration curve obtained using standard solutions at 0.01 to 5 μM. The amount of each compound in the mitochondrial and cytosolic fractions was expressed as pmol/mg proteins.

### Cytotoxicity and Viability

The extracellular release of LDH, considered an index of cell damage and necrosis, was measured spectrophotometrically, as reported (Riganti et al., 2013b). The extracellular medium was centrifuged at 12,000 × *g* for 15 min to pellet cellular debris, the cells were washed with fresh medium, detached with 0.01% trypsin/EDTA, re-suspended in 0.2 ml of 82.3 mM triethanolamine phosphate-HCl (pH 7.6), and sonicated on ice with two 10-sec bursts. LDH activity was measured in the extracellular medium and in the cell lysate: 50 μl of extracellular medium or 5 μl of cell lysate were incubated at 37°C with 5 mM NADH. The reaction was started by adding 20 mM pyruvic acid and was monitored for 6 min, measuring absorbance at 340 nm using a Synergy HT Multi-Detection Microplate Reader (Bio-Tek Instruments, Winoosky, MT, United States). The reaction kinetics were linear throughout the time of measurement. Both intracellular and extracellular enzyme activities were expressed as μmol NADH oxidized/min/dish. The results were expressed as percentage of extracellular LDH versus total (intracellular plus extracellular) LDH. Cell viability was measured by the neutral red staining method, as previously reported (Riganti et al., 2015b). The absorbance of untreated cells was considered as 100% viability; the results were expressed as percentage of viable cells versus untreated cells. IC_50_ was calculated with the CompuSyn software^1^.

### Mitochondrial Mass and Mitochondria Biogenesis

The amount of proteins in mitochondrial extracts, normalized to the total amount of cellular proteins, was considered an index of mitochondrial mass (Jean et al., 2015). Mitochondria biogenesis was evaluated by measuring the expressions of subunit I of COX-I, which is encoded by mitochondrial DNA, and of the SDH-A of complex II, which is encoded by nuclear DNA, using the MitoBiogenesis^TM^ In-Cell ELISA Kit (Abcam), following the manufacturer’s instructions. The unit (U) of each protein/mg mitochondrial proteins were calculated. Results were expressed as COX-1/SDH-A ratio.

### Confocal Microscope Analysis

0.5 × 10^5^ cells were grown on sterile glass coverslips. 200 nM MitoTracker^®^ Green FM (Invitrogen Life Technologies, Milano, Italy) was add for 45 min. Cells were rinsed with PBS, fixed with 3.7% w/v paraformaldehyde (diluted in PBS) for 5 min, washed three times with PBS then the slides were mounted with 10 μl of Gel Mount Aqueous Mounting. The samples were analyzed with an Olympus FV300 laser scanning confocal microscope equipped with a Blue Argon (488 nm) laser, a Green Helium Neon (543 nm) laser, and FluoView 300 software (Olympus Biosystems, Hamburg, Germany). For each experimental conditions, 10 fields were examined.

### Electron Transport Chain

The electron transport between complexes I and III was measured in mitochondrial extracts, immediately after extract thawing, as detailed earlier (Jean et al., 2015). In particular, 10 μl of not-sonicated mitochondrial samples were re-suspended in 0.59 ml buffer C (5 mM KH_2_PO_4_, 5 mM MgCl_2_, 5% w/v BSA) and transferred into a quartz spectrophotometer cuvette. Then 0.38 ml buffer D (25% w/v saponin, 50 mM KH_2_PO_4_, 5 mM MgCl_2_, 5% w/v BSA, 0.12 mM cytochrome c-oxidized form, 0.2 mM NaN_3_) was added for 5 min at room temperature. The reaction was started with 0.15 mM NADH and was followed for 5 min, reading the absorbance at 550 nm by a Lambda 3 spectrophotometer (PerkinElmer, Waltham, MA, United States). The results were expressed as nmoles reduced cytochrome c/min/mg mitochondrial proteins. As reported earlier (Wibom et al., 2002), one cycle of freezing and thawing, following by the addition of 25% w/v saponin, did not destroy mitochondrial membrane structure and integrity and allowed to measure the maximal metabolic and redox capacity of mitochondria. Therefore, this procedure was adopted to evaluate the electron transport chain activity, the ATP and ROS levels, the mitochondrial depolarization.

### Mitochondrial ATP

The amount of ATP was measured on 20 μg of mitochondrial extracts with the ATP Bioluminescent Assay Kit (FL-AA, Sigma Chemical Co.). Data were converted into nmoles/mg mitochondrial proteins, using a calibration curve previously set.

### Mitochondrial ROS Measurement

The amount of ROS in mitochondrial extracts was measured fluorimetrically by incubating cell suspension at 37°C for 10 min with 10 μM of MitoSOX Red probe (Invitrogen Life Technologies), as per manufacturer’s instructions. Results were expressed as nmoles/mg mitochondrial proteins, using a calibration curve previously set with serial dilution of H_2_O_2_.

### Mitochondrial Depolarization

Mitochondrial depolarization was measured by staining cells with the fluorescent probe JC-1 (Biotium Inc., Hayward, CA, United States), as described (Riganti et al., 2015a). The red fluorescence, index of polarized mitochondria, was detected at 550 nm (λ excitation) and 600 nm (λ emission); the green fluorescence, index of depolarized and damaged mitochondria, was detected at 485 nm (λ excitation) and 535 nm (λ emission). The fluorescence units were used to calculate the percentage of green-fluorescent versus red-fluorescent mitochondria.

### Caspase Activity

The activity of caspase 9 and 3 was measured by incubating 20 μg cell lysates with the respective fluorogenic substrates Ac-LEHD-AMC (LEHD-AMC) or DEVD-AMC (DEVD-AMC), as reported (Pinzón-Daza et al., 2012). Results were expressed as nmoles AMC/mg protein.

### Cell Cycle Analysis

1 × 10**^4^** cells were harvested, washed with PBS, treated with 0.25 mg/ml RNAse and stained for 15 min with 50 μg/ml propidium iodide. Cell cycle distribution was analyzed by Guava^®^ easyCyte flow cytometer (Millipore, Billerica, MA, United States), using the InCyte software (Millipore).

### Statistical Analysis

All data in text and figures are provided as means+SD. The results were analyzed by a one-way ANOVA and Tukey’s test. *p* < 0.05 was considered significant.

## Results

### Synthesis of TTP Derivatives

**FIGURE 2:**
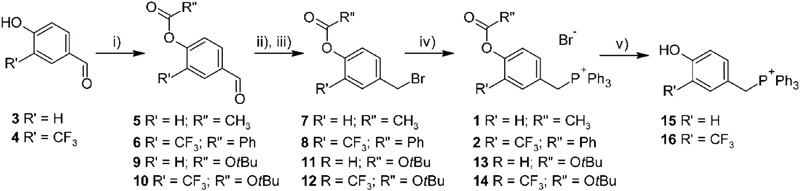
Synthesis of designed TPP-derivatives and TPP-substituted phenols. Reagents and conditions: (i) R”COCl, Et_3_N, CH_2_Cl_2_, 0°C, or Boc_2_O, DMAP, CH_2_Cl_2_, 0 °C; (ii) NaBH_4_, MeOH, 0 °C; (iii) NBS, PPh_3_, CH_2_Cl_2_, 0 °C; (iv) PPh_3_, toluene, reflux; (v) HBr 48% in MeOH, rt.

TPP salts were obtained as described in **Figure 2**. 4-Hydroxybenzaldehydes (**3, 4**) were acylated using acetyl chloride or benzoyl chloride, giving corresponding 4-acyloxybenzaldehydes (**5, 6**). The subsequent reduction of aldehyde with NaBH_4_, followed by the treating with NBS and Ph_3_P, gave benzyl bromide derivatives **7** and **8**. Finally, alkylation of triphenylphosphine in toluene solution afforded desired TPP-derivatives **1** and **2**.

References phenols were obtained following the similar synthetic strategy, using tert-butyl carbonate as a protecting group. The deprotection of tert-butyloxycarbonyl derivatives by action of HBr 48% in methanol afforded 4-hydroxybenzyl TPP bromides **15** and **16**.

### Lipophilicity Measurements

The partition coefficient (log P) of compounds **1** and **2**, as well as their parent phenols **15** and **16**, were measured in n-octanol/PBS system using shake-flask techniques (**Table 1**). All the compounds were quite lipophilic despite the presence of cationic TPP moieties. As expected, the log *P*-values of phenolic esters **1** and **2** were higher with respect to the parent phenols **15** and **16**, but these differences are not high enough to hypothesize the partial ionization of hydroxyl group at pH 7.4. Log *P*-values are close to the optimal range for the passive membrane penetration that should permit good intracellular accumulation of our derivatives.

**Table 1:** ^a^Lipophilicity measurement of phenol TPP-derivatives.

Compound	log P (±*SD*)
1	0.64 (± 0.09)
2	3.15 (± 0.08)
15	0.50 (± 0.06)
16	1.02 (± 0.06)

*^*a*^Each log *P*-value is an average of at least six measurement.*

### Phenol TPP-Derivatives Are Effective Against a Broad Spectrum of Tumor Cells

**FIGURE 3:**
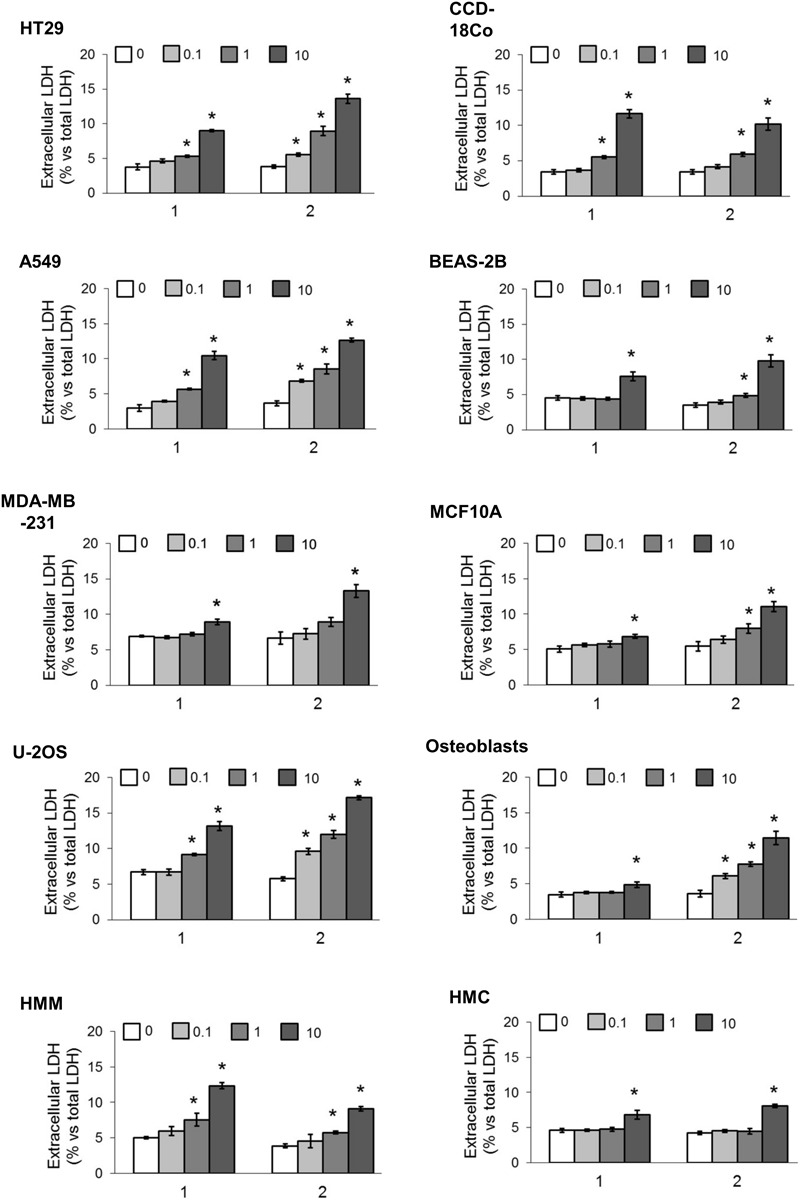
Cytotoxicity in different cancer cell lines and normal cells. Cells were incubated 24 h in fresh medium (0) or with increasing concentrations (0.1, 1, and 10 μM) of **1** and **2**. The release of LDH was measured spectrophotometrically in duplicates. Data are means ± SD (*n* = 3). ^∗^*p* < 0.01 vs. cells grown in fresh medium.

**FIGURE 4:**
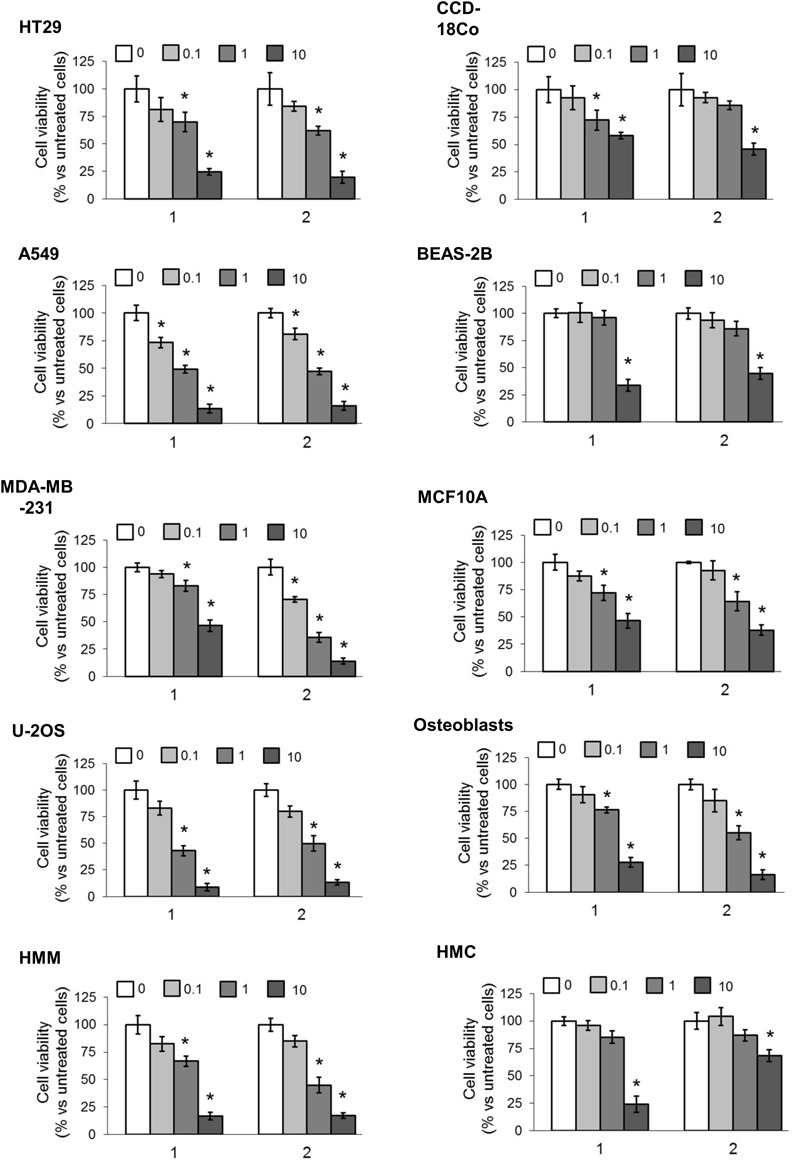
Effects of phenolic TPP-derivatives on the viability of cancer cell lines and normal cells. Cells were incubated 72 h in fresh medium (0) or with increasing concentrations (0.1, 1, and 10 μM) of **1** and **2**. Cell viability was measured spectrophotometrically in quadruplicates. Data are means ± SD (*n* = 3). ^∗^*p* < 0.05 vs. cells grown in fresh medium.

We first screened the cytotoxic effects of different concentrations of our TPP-derivatives, measured as release of LDH in the extracellular medium after 24 h. We analyzed a panel of human cancer cell lines of different histological origin and normal cells of the same organ. As shown in **Figure 3**, both compounds (**1** and **2**) induced a dose-dependent increase of extracellular LDH, either in cancer cell lines and normal cells. Consistently, the cell viability after prolonged exposure (72 h) was dose-dependently reduced (**Figure 4**).

To obtain a most precise quantitative measure of the differential cytotoxicity exerted by compound **1** and **2** in cancer cell lines and normal cells, and to better compare the products, we calculated the IC_50_ for these TPP-derivatives in all the cell lines examined (**Table 2**). Notwithstanding the great variability among each cell line, in all cancer cell lines the IC_50_ for both compounds was below 10 μM. With the exception of the breast cancer/mammary epithelium pair (i.e., MDA-MB-231/MCF10A cells) for compound **1**, the IC_50_ was lower in cancer cell lines than in normal cells for both compounds. In lung cancer A549 cells and in osteosarcoma U-2OS cells, the IC_50_ was below 1 μM, a value in line with several chemotherapeutic drugs. The ratio between the IC_50_ in BEAS-2B cells and A549 cells was 16.97 for compound **1** and 13.38 for compound **2**, respectively. The ratio between the IC_50_ in osteoblasts and U-2OS cells was 14.36 for compound **1** and 5.41 for compound **2**, respectively. Overall, these results confirmed that compounds **1** and **2** showed a generally higher cytotoxicity against cancer cell lines than against normal cells. According to the IC_50_ values, the best cancer cell models, candidate to be treated with the TPP-derivatives were lung cancer and osteosarcoma. Since the lung cancer/normal lung epithelium pair displayed the highest differences between the IC_50_ for both compounds, we focused on this model to investigate the mechanisms of this preferential cytotoxicity.

**Table 2:** IC_50_ (μM) of phenol TPP-derivatives.

Cell line	IC_50_ (μM) of 1	IC_50_ (μM) of 2
HT29	4.45 ± 0.78*	3.81 ± 0.47*
CCD-18Co	18.21 ± 1.29	7.89 ± 0.36
A549	0.31 ± 0.09*	0.55 ± 0.07*
BEAS-2B	5.26 ± 0.25	7.36 ± 0.45
MDA-MB-231	8.32 ± 1.25	0.34 ± 0.11*
MCF10A	7.52 ± 0.43	6.32 ± 0.41
U-2OS	0.45 ± 0.16*	0.63 ± 0.10*
Osteoblasts	6.46 ± 0.83	3.41 ± 0.73
HMM	4.39 ± 0.35*	0.82 ± 0.07*
HMC	7.37 ± 0.89	39.09 ± 4.34

*1 × 10^*4*^ cells were seeded in quadruplicate in 96-well plates, treated at scalar concentrations (from 10^-*10*^ to 10^-*3*^M) of each compounds for 72 h, then stained with neutral red solution. IC_50_ was calculated with the CompuSyn software. Data are means ± SD (*n* = 4). Cancer cell lines (HT29, A549, MDA-MB-231, U-2OS, HMM) vs. normal cells of the same organ (CCD-18Co, BEAS-2B, MCF10A, HMC): ^∗^*p* < 0.05.*

### Phenolic Esters TPP-Derivatives Accumulate in Mitochondria and Are Hydrolyzed to the Corresponding Phenols

As shown in the **Supplementary Figure S2**, TPMP (i.e., TPP moiety only) did not reduce viability of A549 and BEAS-2B cells, suggesting that cytotoxic effect is due to the TPP-derivatives **1** and **2**.

Since TPP moiety is a well-known cation crossing the mitochondrial membranes (Rideout et al., 1989; Bergeron et al., 2009), we first measured the intra-mitochondrial accumulation of these compounds.

**FIGURE 5:**
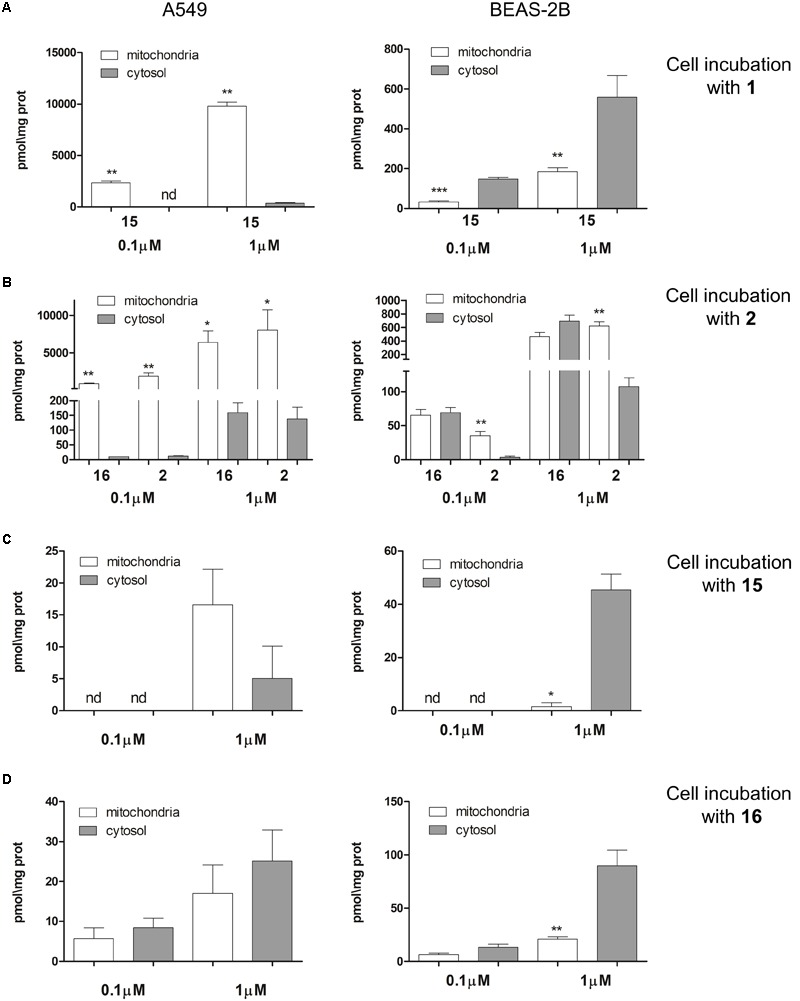
Intra-mitochondrial and cytosolic accumulation of TPP-derivatives. **(A)** A549 (Left) and BEAS-2B (Right) cells were incubated 4 h with two different concentrations (0.1 and 1 μM) of **1**. **(B)** A549 (Left) and BEAS-2B (Right) cells were incubated 4 h with two different concentrations (0.1 and 1 μM) of **2**. **(C)** A549 (Left) and BEAS-2B (Right) cells were incubated 4 h with two different concentrations (0.1 and 1 μM) of **15**. **(D)** A549 (Left) and BEAS-2B (Right) cells were incubated 4 h with two different concentrations (0.1 and 1 μM) of **16**. The concentration of **1, 2, 15**, and **16** in mitochondrial and cytosolic fractions were measured using LC-MS techniques. Data are means ± SD (*n* = 3). ^∗^*p* < 0.01: mitochondria vs. cytosol. ^∗∗^*p* < 0.001: A549 vs. BEAS-2B in all the corresponding conditions **(A,B)**; ^∗^*p* < 0.05: A549 vs. BEAS-2B in all the corresponding conditions **(C)**; ^∗^*p* < 0.05: A549 vs. BEAS-2B (cytosolic fraction, 1 μM conventration **(D)**.

The intra-mitochondrial accumulation of esters **1** and **2** as well as their parent phenols **15** and **16** were evaluated using LC-MS technique, by incubating A549 and BEAS-2B cells with 0.1 and 1 μM solutions as detailed in the Materials and Methods section. After a 4 h incubation, the cellular mitochondrial and cytosolic fractions were separated and the amount of **1, 2, 15**, and **16** in the two fractions was measured by UPLC with mass detector. As shown in **Figure 5** both TPP esters **1** and **2** penetrate in good extent in A549 cells (**Figures 5A,B**, Left), while their concentration in BEAS-2B cells was at least ten fold lower (**Figures 5A,B**, Right), in line with their toxicity profile. Only phenol derivative **15** was detected after incubation with **1** (**Figure 5A**): this result confirms the accumulation of compound **1** with a further fast hydrolysis into phenol **15**. The cytosolic concentration of **15** after incubation with 0.1 μM of **1** was below detection limit, while mitochondrial accumulation was easily detectable. Even after incubation at 1 μM of **1** the cytosolic concentration of **15** was very low. The derivative **2** has a bit different behavior: benzoic ester seems to be more stable with respect to acetate, though both benzoate **2** and corresponding phenol **16** were detected after 4 h of incubation with **2** (**Figure 5B**). Also in this case the compounds accumulate almost exclusively at mitochondrial level, and A549 cells accumulated significantly more TPP-derivatives than BEAS-2B cells.

To verify whether also **15** and **16** entered into the cells, we directly incubated A549 and BEAS-2B cells with these two phenol TPP-derivatives. The intracellular concentration found was very low in both cell lines for either **15 (Figure 5C)** and **16** (**Figure 5D**). Such low intracellular accumulation could explain the lack of toxicity of **15** and **16**: indeed compounds **15** and **16** did not increase the release of LDH (**Supplementary Figure S3A**) nor reduced cell viability (**Supplementary Figure S3B**) when incubated on A549 and BEAS-2B cells. These results exclude that the presence of compounds **15** and **16** in the extracellular environment induce cytotoxicity; however, we cannot exclude *a priori* that they can elicit toxicity once generated inside the cell.

### Phenol TPP-Derivatives Impair Mitochondrial Energetic Metabolism and Trigger Mitochondria-Dependent Apoptosis in Tumor Cells

**FIGURE 6:**
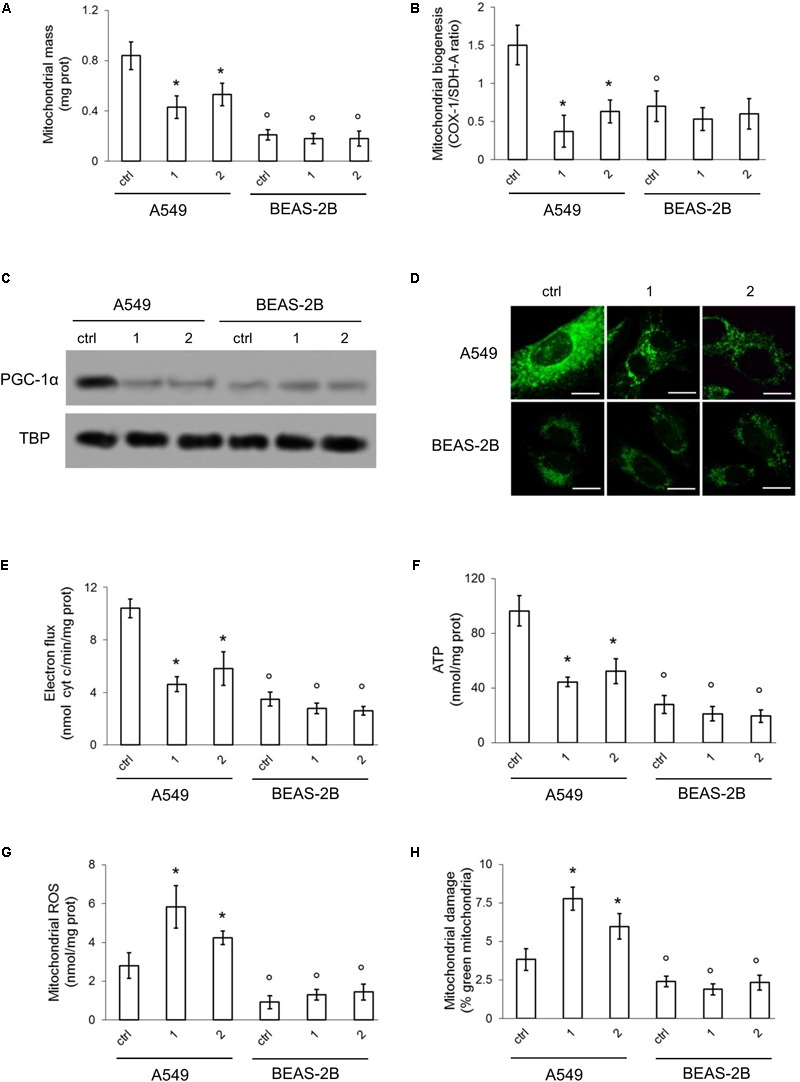
Effects of phenol TPP-derivatives on mitochondria metabolism. A549 and BEAS-2B cells were incubated 4 h in fresh medium (ctrl) or in medium containing 0.1 μM of **1** and **2**. After the separation of mitochondria and cytosol extracts, the following assays were performed. **(A)** Mitochondrial proteins were measured spectrophotometrically in duplicates. Data are means ± SD (*n* = 3). ^∗^*p* < 0.001 vs. A549 ctrl cells; °*p* < 0.001 for BEAS-2B cells vs. the corresponding condition in A549 cells. **(B)** Mitobiogenesis was measured by ELISA, in duplicates. Data are means ± SD (*n* = 3). ^∗^*p* < 0.001 vs. A549 ctrl cells; °*p* < 0.002 for BEAS-2B cells vs. the corresponding condition in A549 cells. **(C)** Immunoblot analysis of PGC-1α and TBP in nuclear extracts. The blot is representative of one out*(Continued)*

Compared to BEAS-2B, A549 cells had a higher mitochondrial mass, measured on the basis of mitochondrial proteins (Jean et al., 2015) (**Figure 6A**) and a higher rate of mitochondrial biogenesis, indicated by the higher ratio between COX-I, which is encoded by mitochondrial DNA, and SDH-A, which is encoded by nuclear DNA (**Figure 6B**). Consistently, the transcription factor PGC-1α, a master regulator of mitobiogenesis (Buondonno et al., 2016), was constitutively translocated into the nucleus (i.e., activated) in A549 cells, but its nuclear level was reduced by compounds **1** and **2**. BEAS-2B cells had lower levels of nuclear PGC-1α, that was unaffected by TPP-derivatives (**Figure 6C**). The staining with Mitotracker Green indicated that A549 cells had higher mitochondrial mass than BEAS-2B. Compounds **1** and **2** reduced the mitochondrial mass in the former but not in the latter. The peri-nuclear distribution of mitochondria was not changed by the TPP-derivatives, that produced, however, a generalized decrease in the mitochondrial amount of A549 cells (**Figure 6D**). To verify whether TPP-derivatives also determined changes in mitochondrial fusion/fission, we measured the expression of mitofusin 1 (*MFN1*) and mitofusin 2 (*MFN2*): no changes in the expression of these genes were detected in A549 and BEAS-2B cells, untreated or treated with TPP-derivatives (**Supplementary Figure S4**), excluding an effect of the compounds on mitochondrial fusion.

The rate of electron transport (**Figure 6E**) and the amount of mitochondrial ATP (**Figure 6F**) were higher in A549 cells. Compounds **1** and **2** produced a significant decrease of these parameters in A549 cells, not in BEAS-2B cells, likely as a consequence of the higher amount of mitochondria, where the compounds were accumulated, and the higher baseline values of mitochondrial energy metabolism of the former.

**FIGURE 7:**
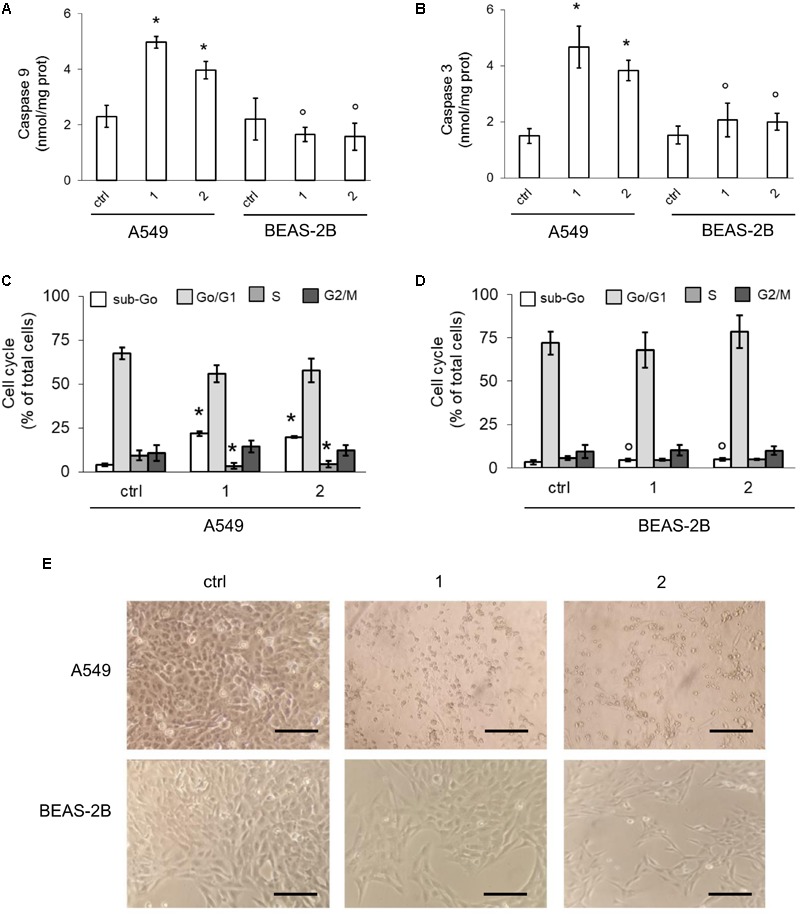
Effects of phenol TPP-derivatives on mitochondria-dependent apoptosis. A549 and BEAS-2B cells were incubated 24 h in fresh medium (ctrl) or in medium containing 0.1 μM of **1** or **2**. **(A,B)** The activity of caspase 9 and 3 was measured fluorimetrically, in duplicates. Data are means ± SD (*n* = 3). For both panels: ^∗^*p* < 0.001 vs. A549 ctrl cells; °*p* < 0.001 for BEAS-2B cells vs. the corresponding condition in A549 cells. (**C,D**) Cell cycle distribution, measured in duplicates. Data are means ± SD (*n* = 3). For both panels: ^∗^*p* < 0.001 vs. A549 ctrl cells: °*p* < 0.001 for BEAS-2B cells *vs*. the corresponding condition in A549 cells. **(E)** Bright field microscope analysis of cells. Magnification: 60 × objective (0.52 numerical aperture); 10 × ocular lens. Bar: 20 μM. The micrographs are representative of three experiments with similar results.

Higher is the electron flux, higher is the possibility to produce ROS because of uncomplete reduction of O_2_, the electrons final acceptor, or redox cycle at complex I and III (Mailloux and Harper, 2012). At high concentrations, ROS damage mitochondria lipids and proteins, open the membrane permeability transition pore and promote the cytosolic release of cytochrome c, which in turn triggers the activation of caspase 9 and 3, producing a necro-apoptotic death (Indran et al., 2011). In keeping with these observations, we detected basally lower levels of mitochondrial ROS (**Figure 6G**) and depolarized/damaged mitochondria (**Figure 6H**) in BEAS-2B compared to A549 cells. In both cell lines, however, the activity of caspase 9 and 3 was similar (**Figures 7A,B**).

In A549 cells compounds **1** and **2** acutely increased the mitochondrial ROS (**Figure 6G**) and the percentage of depolarized mitochondria (**Figure 6H**), triggering a strong activation of caspase 9 and 3 (**Figures 7A,B**). Again, the compounds exerted negligible modifications of ROS (**Figure 6G**) and mitochondrial depolarization (**Figure 6H**) in BEAS-2B cells, where the activity of caspase 3 and 9 remained similar to untreated cells (**Figures 7A,B**).

To prove that mitochondrial ROS were the inducers of cytotoxicity, we measured the viability of cells treated with compounds **1** or **2**, in the presence or absence of MitoTempol, a specific scavenger of ROS of mitochondrial origin (Trnka et al., 2009). As shown in the **Supplementary Figure S5**, MitoTempol alone did not affect A549 and BEAS-2B cells viability, but it significantly prevented the reduction in cell viability induced by compound **1** and **2** in A549 cells. By contrast, no effects were exerted in BEAS-2B cells, where the TPP-derivatives did not increase mitochondrial ROS (**Figure 6G**) and did not lower viability (**Figure 4**).

Consistently with the increased activity of caspases, compounds **1** and **2** increased the percentage of A549 cells in the sub-Go phase, indicating a higher percentage of apoptotic cells. This event was paralleled by a reduced percentage of cells in S-phase, suggesting decreased proliferation (**Figure 7C**). By contrast, no change in cell cycle distribution was noticed in not-transformed BEAS-2B cells (**Figure 7D**), in keeping with the absent activation of caspases. Morphological studies revealed that, compared with untreated cells, A549 cells exposed to compounds **1** or **2** showed a reduced cell number and a clear round-shaped morphology, suggestive of apoptosis (**Figure 7E**). BEAS-2B cells treated with TPP-derivatives only showed a decreased cell density without appreciable morphological signs of apoptosis (**Figure 7E**).

[t] Continuedof three experiments with similar results. **(D)** Confocal analysis of mitochondrial staining with MitoTracker^®^ Green FM. Magnification: 60 × objective (0.52 numerical aperture); 10 × ocular lens. Bar: 10 μM. The micrographs are representative of three experiments with similar results. **(E,F)** The electron transport was measured spectrophotometrically in duplicates. ATP levels in mitochondrial extracts was measured by a chemiluminescence-based assay, in duplicates. Data are means ± SD (*n* = 3). For both panels: ^∗^*p* < 0.001 vs. A549 ctrl cells; °*p* < 0.001 for BEAS-2B cells vs. the corresponding condition in A549 cells. **(G,H)** ROS amount in mitochondrial extracts and mitochondrial damage were measured fluorimetrically, in duplicates. Data are means ± SD (*n* = 3). For both panels: ^∗^*p* < 0.001 vs. A549 ctrl cells; °*p* < 0.001 for BEAS-2B cells *vs*. the corresponding condition in A549 cells. There were no statistically significant differences between untreated (ctrl) and compound **1**/compound **2**-treated BEAS-2B cells for all the parameters analyzed in this figure.

## Discussion

By performing a screening on a panel of human tumor and not-transformed cells, we identified two TPP-phenol derivatives (**1** and **2**) that displayed cytotoxic activity and reduced cell viability of cancer cell lines *in vitro*, in keeping with previous observations reporting antitumor activity of TPP salts (Rideout et al., 1989; Manetta et al., 1996). Both TPP-phenol derivatives were endorsed with cytotoxic activity at low micromolar/submicromolar (0.1–1 μM) concentrations. This efficacy was similar to the antitumor activity of the most effective TPP salts, selected over a library of 40000 candidates (Millard et al., 2010). Of note, the reduced viability was achieved in all the cancer cell lines, independently of the histological origin, although different cancer cell lines exhibited different IC_50_ to the compounds. This finding suggests that the compounds exert their cytotoxic effects exploiting biochemical mechanisms that are common to all cancer cell lines tested. Another key feature of the phenol TPP-derivatives synthesized is that the anti-proliferative effect appeared lower - comparing the same concentration and incubation time - in normal cells.

To investigate the biochemical mechanisms that may explain the cytotoxic efficacy of the phenol TPP-derivatives and their greater potential against tumor cells, we focused on the lung cancer/normal lung epithelium pair, which displayed the highest differences between the cytotoxicity of both compounds.

Since TPP cation is a well-known vector for the mitochondria (Rideout et al., 1989; Bergeron et al., 2009), we first evaluated the intra-mitochondrial accumulation of these compounds, finding that our phenolic esters **1** and **2** accumulate in mitochondria and produce phenols **15** and **16** with different kinetics. Compounds **1** and **2** accumulation in normal cells was significantly lower than in cancer cells.

The high intra-mitochondrial accumulation of our TPP-derivatives prompted us to investigate if they may interfere with the mitochondrial energetic metabolism and redox balance, two processes that – when altered – reduce cancer cell proliferation and trigger apoptosis (Trotta and Chipuk, 2017).

Consistent with experimental observations demonstrating that mitochondria play a key role in sustaining tumor energetic demand (Caino et al., 2015; Trotta and Chipuk, 2017; García-Ledo et al., 2017), A549 cells had a significantly higher mitochondrial mass and energetic metabolism. This phenotype is likely due to the increased mitobiogenesis sustained by the constitutive activation of PGC-1α in A549 cells. By reducing the active PGC-1α, the amount of mitochondria and the ability to synthesize ATP via the electron transport chain, compounds **1** and **2** interfered with the key compartments that provide cancer cells with the necessary fuel. It is generally accepted that the transmembrane potential of normal cells is less negative, suggesting a lower energetic activity of mitochondria in not-tumor tissues (Modica-Napolitano and Aprille, 2001). This finding - that was confirmed by comparing BEAS-2B cells with A549 cells - may explain why the effects of **1** and **2** were negligible in BEAS-2B cells. Since the lower potential of mitochondrial membrane in normal cells theoretically also reduces the delivery of phenol TPP-derivatives into mitochondria of these cells, we might speculate that the absence of metabolic effects in BEAS-2B can be due to the lower mitochondrial accumulation of **1** and **2**. In addition BEAS-2B cells had a lower PGC-1α activation that may result in reduced mitobiogenesis and mitochondrial mass. All these events, strongly reduced by TPP-derivatives in A549 cells, were not modified in BEAS-2B cells.

Whatever the mechanism can be, the higher derangement of mitochondrial metabolism in cancer cells and the absence of effects in normal cells, make phenol TPP- derivatives promising cancer cell-selective compounds, unveiling a property that is uncommon for most chemotherapeutic drugs.

Notwithstanding A549 cells displayed a higher proton motive-force coupled with higher intra-mitochondrial ROS levels, this situation did not induce mitochondrial depolarization or mitochondrial-triggered apoptosis. Cancer cells have high levels of anti-oxidant enzymes, including the mitochondrial superoxide dismutase 2, that may buffer ROS more efficiently than in non-transformed cells (Idelchik et al., 2017). On the other hand, low levels of mitochondrial ROS are known to promote oncogenic pro-survival pathways (Idelchik et al., 2017) and to train cancer cells to a better adaptation to stressing conditions that prevent cell damage, a process known as mitohormesis (Yun and Finkel, 2014). Only a significant increase in ROS levels may overcome anti-oxidant defenses and mitohormetic adaptation, triggering cell death instead of cell survival mechanisms (Idelchik et al., 2017; Yun and Finkel, 2014).

This is indeed what happened in A549 cells treated with **1** and **2**, that increased the amount of intra-mitochondrial ROS and damaged mitochondria, inducing the activation of caspase 9 and 3 and increasing apoptosis. Since MitoTempol, a specific scavenger of mitochondrial ROS, prevented the reduction in viability induced by **1** and **2**, it is likely that the cytotoxic effect of the TPP-derivatives was due to the increase in mitochondrial ROS in this cancer cell line. Again, all these effects were absent in BEAS-2B cells, where the baseline mitochondrial metabolism was lower and not altered by **1** and **2**.

Of note, A549 cells and U-2OS cells, the two cell lines that displayed the higher cytotoxicity exerted by compounds **1** and **2**, had higher values of electron transport chain, compared to HT29 and MDA-MB-231 cells, where the compounds were less effective. Indeed the rate of electron chain transport was 10.41 + 0.72 nmoles reduced cytochrome c/min/mg mitochondrial proteins in A549 cells (**Figure 6C**), 6.58 + 0.51 nmoles reduced cytochrome c/min/mg mitochondrial proteins – corresponding to an oxygen consumption rate of 620 + 44 pmoles/min - in U-2OS cells (Buondonno et al., 2016), 2.37 + 0.45 nmoles reduced cytochrome c/min/mg mitochondrial proteins in HT29 cells (Riganti et al., 2013a), 2.11 + 0.275 nmoles reduced cytochrome c/min/mg mitochondrial proteins in MDA-MB-231 cells (Gazzano et al., 2018). These observations may explain the differential cytotoxic effects of the TPP-derivatives among different cancer cell lines, suggesting that the compounds are more effective against cancer cells with a higher mitochondrial energy metabolism. On the other hand, we cannot exclude that other mechanisms, such as a different intracellular and/or intramitochondrial accumulation of the compounds, may explain the different efficacy in different cell lines. Ongoing screenings at our laboratory on a larger panel of cancer cell lines with different rates of electron transport chain will clarify this point.

Collectively, these results reinforce the evidences that compound **1** and **2** exert a preferential cytotoxic effect in cancer cells. This feature may be due to at least two reasons. First, cancer cells had higher basal mitobiogenesis than normal cells, likely due to the constitutively activation of PGC-1α, and compounds **1** and **2** reduced these events in cancer cells but not in normal cells. Second, the high accumulation of compounds **1** and **2** and their metabolites within mitochondria, impaired the mitochondrial energy metabolism and increased the synthesis of ROS, that induced a mitochondrial damage and triggered the caspase 9/caspase 3-dependent apoptosis.

Despite a reduction in the cell number of BEAS-2B cells, compounds **1** and **2** did not exert significant effects on cell cycle progression, apoptosis or LDH release. Most importantly, the compounds did not affect cell viability of normal cells after 72 h. On the basis of these results, we may hypothesize that TPP-derivatives induced a cytostatic effect at short time in normal cells that are however able to recover and survive. By contrast, compounds **1** and **2** induced an irreversible mitochondrial damage of cancer cells that trigger apoptosis and reduced cell viability. The different targeting of mitochondrial metabolisms in cancer and normal cells may explain such differential behavior. We are aware that the *in vitro* experiments cannot guarantee that the compounds are safe for healthy tissues, since these data can be obtained only by *in vivo* experiments. However, the higher intracellular and intra-mitochondrial accumulation, and the higher derangement of mitochondrial energetic and redox metabolism induced by TPP-derivatives in cancer cells versus normal cells are good premises to study the compounds in preclinical models to ascertain the real safety for healthy tissues and the existence of a suitable therapeutic window, exploitable in clinical settings.

## Conclusion

We developed two phenolic esters TPP-derivatives that have higher cytotoxicity against cancer cells than against normal cells. The reasons of such efficacy and selectivity are the high intra-mitochondrial accumulation of the compounds and their ability to impair a metabolic phenotype of cancer cells, i.e., the high mitochondrial metabolism that is vital to meet the energetic demand of cancer cells. The events induced by phenol TPP-derivatives reproduce what we already observed in cancer cells treated with doxorubicin-derivatives vectorised to mitochondria, that impair mitochondrial energy metabolism, increase intra-mitochondrial ROS, trigger mitochondrial depolarization, and apoptosis (Riganti et al., 2015a; Buondonno et al., 2016). These data suggest that – independently of the structure – drugs that interfere with mitochondrial functions are promising anti-cancer tools.

## Author Contributions

EG, KC, and CR designed the experiments and analyzed the data. EG, LL, BR, JK, SG, and CC performed the experiments. EG, LL, and BR interpreted the results. KC and CR wrote the paper. All authors discussed the results and contributed to the manuscript.

## Conflict of Interest Statement

The authors declare that the research was conducted in the absence of any commercial or financial relationships that could be construed as a potential conflict of interest.

## Footnotes

1 http://www.combosyn.com

## Supplementary Material

FIGURES S1–S5 Synthetic procedures and physicochemical characterization of all the compounds, qRT-PCR method.

## Abbreviations

AMC: 7-amino-4-methylcoumarin
ANOVA: analysis of variance
BSA: bovine serum albumin
COX-I: cytochrome c oxidase/complex IV
HMM: human malignant mesothelioma
HMC: human mesothelial cells
LC-ESI-MS: liquid chromatography-electrospray ionization-tandem mass spectrometry
LDH: lactate dehydrogenase
MitoTempol: (2-(2,2,6,6-Tetramethylpiperidin-1-oxyl-4-ylamino)-2-oxoethyl)triphenylphosphonium chloride
PBS: phosphate buffered saline
ROS: reactive oxygen species
SDH-A: succinate dehydrogenase-A
SDS–PAGE: sodium dodecyl sulfate – polyacrylamide gel electrophoresis
TPMP: methyltriphenylphosphonium chloride
TPP: triphenylphosphonium
