# Supplementary material for: Mitochondrial Delivery of Phenol Substructure Triggers Mitochondrial Depolarization and Apoptosis of Cancer Cells
Source: Front Pharmacol. 2018 Jun 4;9:580. doi: 10.3389/fphar.2018.00580 (PMC5994430; doi:10.3389/fphar.2018.00580)
Supplement: FIGURES S1–S5 — Synthetic procedures and physicochemical characterization of all the compounds, qRT-PCR method. [file Presentation_1.pdf]

Supplementary Material

Mitochondrial delivery of phenol substructure triggers mitochondrial depolarization and apoptosis of cancer cells

Elena Gazzano[1], Loretta Lazzarato[2], Barbara Rolando[2], Joanna Kopecka[1], Stefano Guglielmo[2], Costanzo Costamagna[1], Konstantin Chegaev[2a*], Chiara Riganti[1a*]

*** Correspondence:**

Chiara Riganti: chiara.riganti@unito.it

Konstantin Chegaev: konstantin.chegaev@unito.it

Supplementary experimental section

^{1}H and ^{13}C NMR spectra were recorded on a BrukerAvance 300, at 300 and 75 MHz, respectively, using $SiMe_4$ as internal standard. The following abbreviations indicate peak multiplicity: s = singlet, d = doublet, t = triplet, q = quartet, m = multiplet. ESI spectra were recorded on Micromass Quattro API micro (Waters Corporation, Milford, MA, USA) mass spectrometer. Data were processed using a MassLynxSystem (Waters). Flash column chromatography was performed on silica gel (Merck Kieselgel 60, 230–400 mesh ASTM). The progress of the reactions was followed by thin-layer chromatography (TLC) on 5 × 20 cm plates Merck Kieselgel 60 F_{254}, with a layer thickness of 0.20 mm. Anhydrous sodium sulfate (Na_2SO_4) was used as drying agent for the organic phases. Organic solvents were removed under reduced pressure at 30 °C. Synthetic-purity solvents ethyl acetate (EtOAc), acetone, dichloromethane (DCM), acetonitrile (CH_3CN), methanol (MeOH), diethyl ether (Et_2O), and 40–60 petroleum ether (PE) were used. Dry tetrahydrofuran (THF), was distilled immediately before use from Na and benzophenone under positive N_2 pressure. Dry CH_2Cl_2 was distilled from P_2O_5 and stored on molecular sieves 4Å. Commercial starting materials were purchased from Sigma-Aldrich, Alfa Aesar, and TCI Europe. The purity of final compounds **1**, **2**, **15** and **16** was determined by UPLC analyses. Analyses were performed with an Acquity Ultra Performance LC™, Waters Corporation Milford MA, USA, equipped with BSM, SM, CM and PDA detector. All the chromatographic separations were performed on a Zorbax Eclipse XDB-C18 (5 µm , 150 mm × 4.6 mm) (Agilent Technologies) as a stationary phase. Compounds were eluted with a mobile phase (flow rate, 0.5 mL/min) consisting of A, 0.1% formic acid solution, and B, acetonitrile. The following gradient was used: 0-5 min (A = 50%, B = 50%), 5-7 min (A = 20%, B = 80%), 7-8 min (A = 20%, B = 80%), 8-10 min (A = 50%, B = 50%). Compounds **4**,[1] **7**[2] and **9**[3] were prepared as described elsewhere.

[4-(Acetyloxy)benzyl]triphenylphosphonium bromide (1): a solution of **7** (0.33 g, 1.44 mmol) and PPh_3 (0.58 g, 2.21 mmol) in toluene (15 mL) was heated at reflux for 4h. Then the reaction mixture was cooled to rt and white precipitate was collected by filtration. Product was extensively washed with toluene (3×15 mL) and diethyl ether (3×50 mL) and dried in desiccator. Yield 0.65 g (92%). ^{1}H-NMR (DMSO-d_6) δ ppm: 2.23 (s, 3H, CH_3), 5.25 (d, 2H, J^2_{HP} = 15.4 Hz, CH_2), 7.01 (s, 4H, 4C*H*), 7.65 – 7.78 (m, 12H, 12C*H*), 7.89 – 7.94 (m, 3H, 3C*H*); ^{13}C-NMR (DMSO-d_6) δ ppm: 20.8, 27.5 (d, J^1_{CP} = 47.0 Hz), 117.7 (d, J^1_{CP} = 85.7 Hz), 122.5 (d), 125.4 (d), 130.1 (d), 131.9 (d), 134.0 (d), 135.2 (d), 150.4 (d), 169.0 (d). MS (ESI$^+$) *m/z* 411 $[M]^+$. UPLC: t_R = 3.79 min, purity 100%.

4-Formyl-2-(trifluoromethyl)phenyl benzoate (6): to a solution of **4** (0.50 g, 2.63 mmol) in dry CH_2Cl_2 (15 mL), placed in ice bath Et_3N (0.55 mL, 3.95 mmol) was added in one portion followed by benzoylchloride (0.37 mL, 3.16 mmol). The cooling bath was removed and the reaction was allowed to reach rt. After 2h reaction mixture was diluted with CH_2Cl_2 (50 mL) and the organic phase was washed with 1N HCl solution (25 mL), $NaHCO_3$ sat. sol. (25 mL) and brine. The organic extract was dried and evaporated. Obtained oil was purified by flash chromatography (eluent 95/5 PE/acetone) to give the title compound as a colorless oil. Yield 0.42 g (55%). ^{1}H-NMR ($CDCl_3$) δ ppm: 7.52 – 7.57 (m, 2H, 2C*H*), 7.65 – 7.71 (m, 2H, 2C*H*), 8.14 – 8.26 (m, 4H, 4C*H*), 10.06 (s, 1H, CO*H*); ^{13}C-NMR ($CDCl_3$) δ ppm: 122.4 (q, J^1_{CF} = 273.1 Hz), 123.9 (q, J^2_{CF} = 32.6 Hz), 125.3, 128.0, 128.4 (q, J^3_{CF} = 5.0 Hz), 128.8, 130.4, 133.6, 134.0, 134.4, 152.9 (q), 163.7, 189.6. MS (ESI$^+$) *m/z* 295 $[M+H]^+$.

4-Bromomethyl-2-(trifluoromethyl)phenyl benzoate (8): to a solution of **6** (0.36 g, 1.22 mmol) in dry THF (15 mL), placed in ice-salt bath $NaBH_4$ (70 mg, 1.83 mmol) was added in one portion. Reaction mixture was stirred at -10 °C for 1h, then was diluted with EtOAc (50 mL) and organic phase was washed with H_2O (25 mL), brine, dried and evaporated. Obtained brownish solid was used

further without purification. Solid was dissolved in dry CH_2Cl_2 (15 mL) and the obtained solution was placed in ice-salt bath. PPh_3 (0.19 g, 0.70 mmol) was added followed by NBS (0.16 g, 0.88 mmol). The cooling bath was removed and the reaction was allowed to reach rt. After 2 h the solvent was removed and product was purified by flash chromatography (eluent 8/2 PE/CH_2Cl_2) to give the title compound as a white solid. Yield 0.24 g (67%). ^{1}H-NMR ($CDCl_3$) δ ppm: 4.54 (s, 2H, C*H*$_2$), 7.43 (d, 1H, C*H*), 7.55 (t, 2H, 2C*H*), 7.66 – 7.76 (m, 3H, 3C*H*), 8.20 – 8.23 (m, 2H, 2C*H*); ^{13}C-NMR ($CDCl_3$) δ ppm: 31.4, 122.6 (q, J^1_{CF} = 273.1 Hz), 123.3 (q, J^2_{CF} = 32.0 Hz), 124.9, 127.6 (q, J^3_{CF} = 5.0 Hz), 128.5, 128.7, 130.4, 133.6, 134.1, 135.8, 148.2 (q), 164.3. MS (ESI$^+$) *m/z* 359/361 $[M+H]^+$.

[4-(Benzoyloxy)-3-trifluoromethylbenzyl]triphenylphosphonium bromide (2): a solution of **8** (0.15 g, 0.43 mmol) and PPh_3 (0.17 g, 0.64 mmol) in toluene (15 mL) was heated at reflux for 4h. Then the reaction mixture was cooled to rt and white precipitate was collected by filtration. Product was extensively washed with toluene (3×15 mL) and diethyl ether (3×50 mL) and dried in desiccator. Yield 0.23 g (88%). ^{1}H-NMR (DMSO-d_6) δ ppm: 5.45 (d, 2H, J^2_{HP} = 15.9 Hz, C*H*$_2$), 7.26 (m, 1H, C*H*), 7.56 (s, 2H, 2C*H*), 7.63 (t, 2H, 2C*H*), 7.72 – 7.79 (m, 14H, 14C*H*), 7.91 – 7.96 (m, 3H, 3C*H*), 8.08 (d, 1H, 1C*H*); ^{13}C-NMR (DMSO-d_6) δ ppm: 27.3 (d, J^1_{CP} = 47.6 Hz), 117.2 (d, J^1_{CP} = 85.7 Hz), 121.6 (dq, J^2_{CF} = 31.5 Hz, J^4_{CP} = 3.7 Hz), 122.4 (dq, J^1_{CF} = 272.5 Hz, J^5_{CP} = 3.3 Hz), 125.8 (q), 127.1 (d), 127.8, 129.3, 129.4 (m), 129.8, 130.2 (d), 134.1 (d), 134.7, 135.3, 136.1 (d), 147.7 (d), 163.8 (d). MS (ESI$^+$) *m/z* 541 $[M]^+$. UPLC: t_R = 8.58 min, purity 100%.

4-(Bromomethyl)phenyl *tert*-butyl carbonate (11): to a solution of **9** (1.16 g, 5.22 mmol) in dry THF (20 mL), placed in ice-salt bath $NaBH_4$ (0.24 g, 7.84 mmol) was added in one portion. Reaction mixture was stirred at -10 °C for 1h, then was diluted with EtOAc (50 mL) and organic phase was washed with H_2O (25 mL), brine, dried and evaporated. Obtained oil was used further without purification. Oil (0.12 g, 0.54 mmol) was dissolved in dry CH_2Cl_2 (1o mL) and the obtained solution was placed in ice-salt bath. PPh_3 (0.17 g, 0.64 mmol) was added followed by NBS (0.14 g, 0.80 mmol). The cooling bath was removed and the reaction was allowed to reach rt. After 2 h the solvent was removed and product was purified by flash chromatography (eluent 8/2 PE/CH_2Cl_2) to give the title compound as a colorless oil. Overall yield 42%. ^{1}H-NMR ($CDCl_3$) δ ppm: 1.59 (s, 3H, C*H*$_3$), 4.52 (s, 2H, C*H*$_2$), 7.18 (d, 2H, 2C*H*), 7.43 (d, 2H, 2C*H*); ^{13}C-NMR ($CDCl_3$) δ ppm: 27.8, 32.8, 83.9, 121.8, 130.3, 135.4, 151.0, 151.8. MS (ESI$^+$) *m/z* 304/306 $[M+NH_4^+]^+$, 248/250 $[M\text{-}t\text{Bu}+NH_4^+]^+$.

4-Hydroxybenzyltriphenylphosphonium bromide (15): a solution of **11** (0.10 g, 0.35 mmol) and PPh_3 (0.14 g, 0.52 mmol) in toluene (10 mL) was heated at reflux for 4h. Then the reaction mixture was cooled to rt and white precipitate was collected by filtration. Product was extensively washed with toluene (3×5 mL) and diethyl ether (3×10 mL) and dried in desiccator. Obtained solid was dissolved in MeOH (5 mL) and HBr 48% solution (0.5 mL) was added at rt. The reaction mixture was stirred for 48 h, then poured into NaBr sat. sol. (15 mL) and extracted with CH_2Cl_2 (50 mL). Organic phase was washed with NaBr sat. sol. (15 mL), dried and evaporated. Obtained white solid was suspended in Et_2O (25 mL), filtered, washed with Et_2O (25 mL) and desiccated. Overall yield 64%. ^{1}H-NMR (DMSO-d_6) δ ppm: 5.03 (d, 2H, J^2_{HP} = 14.8 Hz, C*H*$_2$), 6.60 (m, 2H, 2C*H*), 6.76 (m, 2H, 2C*H*), 7.64-7.65 (m, 12H, 12C*H*), 7.89 – 7.91 (m, 3H, 3C*H*), 9.62 (s, 1H, O*H*); ^{13}C-NMR (DMSO-d_6) δ ppm: 27.6 (d, J^1_{CP} = 44.8 Hz), 115.7 (d), 117.1 (d), 118.0 (d, J^1_{CP} = 85.3 Hz), 130.1 (d), 132.0 (d), 134.0 (d), 135.0, 157.4. MS (ESI$^+$) *m/z* 369 $[M]^+$. UPLC: t_R = 3.22 min, purity 97.5%.

***tert*-Butyl 4-formyl-2-(trifluoromethyl)phenyl carbonate (10)**: to a suspension of **4** (1.26 g, 6.56 mmol) in dry CH_2Cl_2 (30 mL) Boc_2O (2.94 g, 13.5 mmol) was added followed by DMAP (25 mg, 0.20 mmol). Reaction mixture became clear and was stirred at rt for 24h, than imidazole (1.0 g, 14.7 mmol) was added. Reaction was stirred for additional 10 min, than poured into 1% HCl solution. Organic phase was separated and washed with 1% HCl solution (2×50 mL), NaOH 0.1 N sol. (20 mL), brine, dried and evaporated. Obtained oil was purified by flash chromatography (eluent 7/3

PE/EtOAc) to give the title compound as a colorless oil. Yield 1.44 g (76%). ^{1}H-NMR ($CDCl_3$) δ ppm: 1.57 (s, 9H, 3CH_3), 7.52 (d, 1H, C*H*), 8.11 – 8.14 (m, 1H, C*H*), 8.20 (s, 1H, C*H*), 10.04 (s, 1H, CO*H*); ^{13}C-NMR ($CDCl_3$) δ ppm: 27.5, 85.3, 122.2 (q, J^{1}_{CF} = 273.1 Hz), 124.2 (q, J^{2}_{CF} = 34.3 Hz), 125.2, 128.0, 128.6 (q), 133.8, 134.0, 150.3, 152.8.

4-(Bromomethyl)-2-(trifluoromethyl)phenyl *tert*-butyl carbonate (12): to a solution of **10** (0.21 g, 0.72 mmol) in dry THF (20 mL), placed in ice-salt bath $NaBH_4$ (41 mg, 1.08 mmol) was added in one portion. Reaction mixture was stirred at -10 °C for 1h, then was diluted with EtOAc (50 mL) and organic phase was washed with H_2O (25 mL), brine, dried and evaporated. Obtained oil was used further without purification. Oil (0.13 g, 0.43 mmol) was dissolved in dry CH_2Cl_2 (10 mL) and the obtained solution was placed in ice-salt bath. PPh_3 (0.14 g, 0.51 mmol) was added followed by NBS (0.11 g, 0.60 mmol). The cooling bath was removed and the reaction was allowed to reach rt. After 2 h the solvent was removed and product was purified by flash chromatography (eluent 8/2 PE/CH_2Cl_2) to give the title compound as a colorless oil. Overall yield 40%. ^{1}H-NMR ($CDCl_3$) δ ppm: 1.56 (s, 9H, 3CH_3), 4.49 (s, 2H, CH_2), 7.28 (d, 1H, C*H*), 7.59 – 7.63 (m, 1H, C*H*), 7.68 – 7.69 (m, 1H, C*H*); ^{13}C-NMR ($CDCl_3$) δ ppm: 27.5, 31.2, 84.7, 122.5 (q, J^{1}_{CF} = 273.1 Hz), 123.4 (q, J^{2}_{CF} = 31.5 Hz), 124.8, 127.5 (q), 133.6, 136.0, 148.2, 151.1. MS (ESI^+) *m/z* 372/374 $[M+NH_4^+]^+$.

[4-Hydroxy-3-trifluoromethylbenzyl]triphenylphosphonium bromide (16): a solution of **12** (0.45 g, 1.27 mmol) and PPh_3 (0.36 g, 1.39 mmol) in toluene (15 mL) was heated at reflux for 4h. Then the reaction mixture was cooled to rt and white precipitate was collected by filtration. Product was extensively washed with toluene (3×5 mL) and diethyl ether (3×10 mL) and dried in desiccator. Obtained solid was dissolved in MeOH (5 mL) and HBr 48% solution (1 mL) was added at rt. The reaction mixture was stirred for 48 h, white precipitate formed and was collected by filtration. Obtained white solid was washed with cold H_2O (15 mL) and desiccated. Overall yield 44%. ^{1}H-NMR (DMSO-d_6) δ ppm: 5.15 (d, 2H, J^{2}_{HP} = 14.8 Hz, CH_2), 6.91 – 6.93 (m, 2H, 2C*H*), 7.14 (d, 1H, 1C*H*), 7.67 – 7.77 (m, 12H, 12C*H*), 7.89 – 7.92 (m, 3H, 2C*H*), 10.82 (s, 1H, O*H*); ^{13}C-NMR (DMSO-d_6) δ ppm: 27.2 (d, J^{1}_{CP} = 46.2 Hz), 155.5 (d), 117.4, 117.5, 117.6 (d, J^{1}_{CP} = 85.2 Hz), 123.5 (q, J^{1}_{CF} = 273.1 Hz), 129.3 (m), 130.1 (d), 134.1 (d), 135.2, 135.9 (m), 155.8. MS (ESI^+) *m/z* 437 $[M]^+$. UPLC: t_R = 3.86 min, purity 100%.

Quantitative Real Time-PCR (qRT-PCR).

RNA was extracted and reverse-transcribed using the iScript™ cDNA Synthesis Kit (Bio-Rad Laboratories, Hercules, CA). qRT-PCR was performed using IQ™ SYBR Green Supermix (Bio-Rad Laboratories). The same cDNA preparation was used to measure the genes of interests, mitofusin 1 (*MFN1*) and mitofusin 2 (*MFN2*), and the housekeeping gene *S14*. Primer sequences were designed using qPrimerDepot software (http://primerdepot.nci.nih.gov/). Relative gene expression levels were calculated using Gene Expression Quantitation software (Bio-Rad Laboratories).

Supplementary References

1. Mahaney, P.E.; Krim, L.D.; Jenkins, D.J. Preparation of N-arylalkyl N-heterocyclic derivatives as monoamine reuptake modulators, WO 2005037279 A1, **2005**.

2. Daily, W.; Hawkins, E.; Klaubert, D.; Liu, J.; Meisenheimer, P.; Scurria, M.; Shultz, J.W.; Unch, J.; Wood, K.V.; Zhou, W.; Valley, M.P.; Cali, J.J. Luminogenic and fluorogenic compounds and methods to detect molecules or conditions, WO 2006130551 A2, **2006**.

3. Seki, H.; Xue, S.; Pellett, S.; Silhar, P.; Johnson, E.A.; Janda, K.D. Cellular protection of SNAP-25 against botulinum neurotoxin/A: inhibition of thioredoxin reductase through a suicide substrate mechanism, *J. Am. Chem. Soc.* **2016**, *138 (17)*, 5568-5575.

Supplementary Figures

Supplementary Figure 1

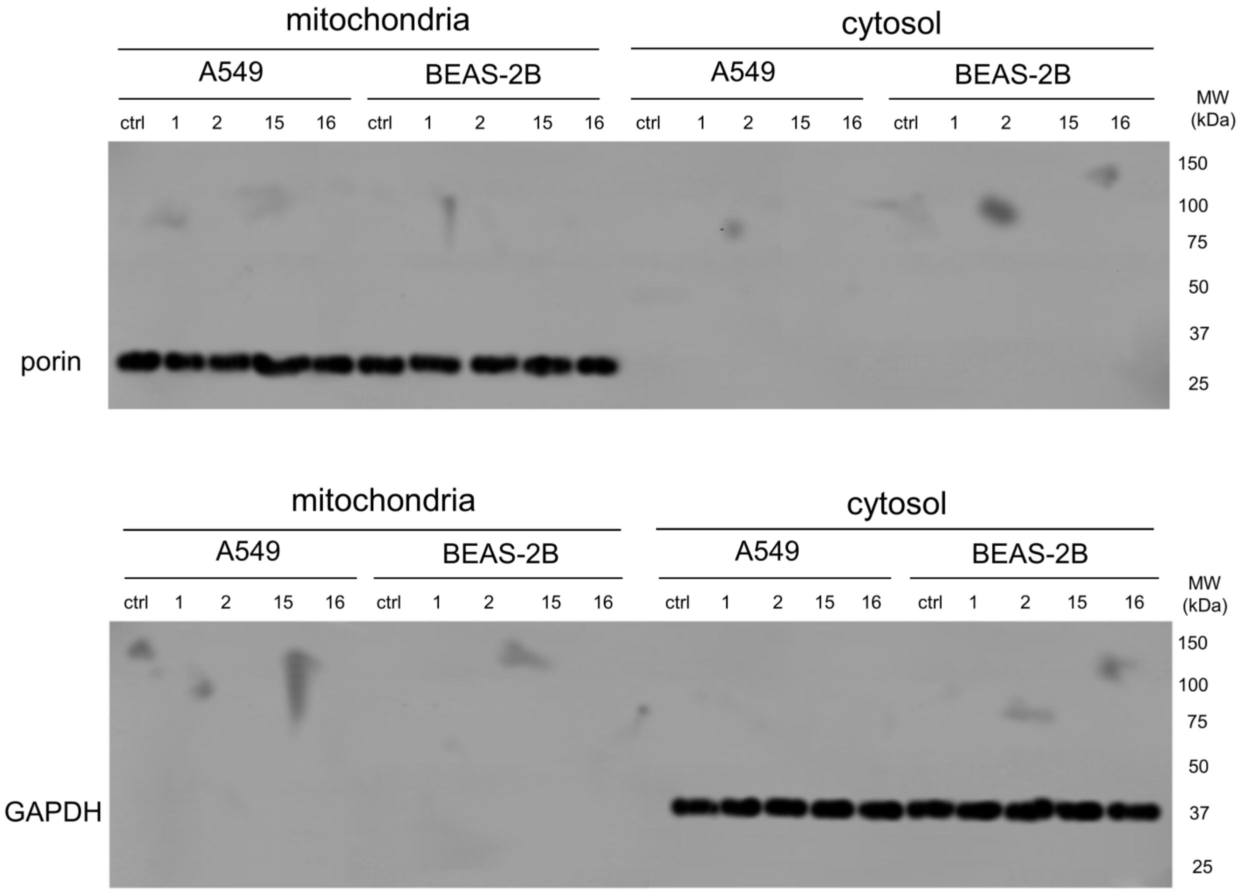

Supplementary Figure 1. Detection of mitochondrial and cytosolic markers by immunoblotting. A549 and BEAS-2B cells were grown in fresh medium (ctrl) or incubated with 1 µM of **1**, **2**, **15** and **16**. After 4 h, mitochondria and cytosol were separated as reported in the MATERIALS AND METHODS section, resolved by SDS-PAGE and probed with antibodies specific for porin, a marker of mitochondria, and glyceraldehyde 3-phosphate dehydrogenase (GAPDH), a cytosolic marker. The figure is representative of the procedure followed to verify the absence of cytosolic contamination in the mitochondrial fractions, and *vice-versa*, in all the mitochondria-cytosol separations performed. MW: molecular weight.

Supplementary Figure 2

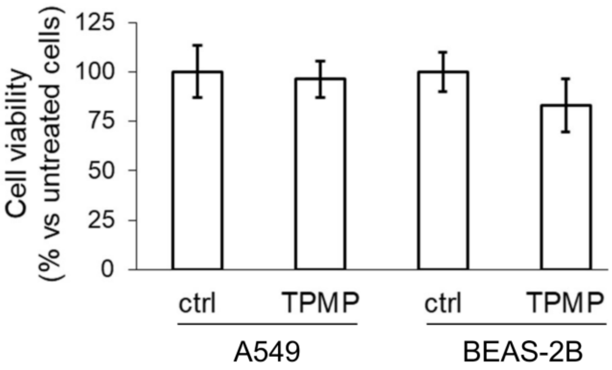

Supplementary Figure 2. Effects of methyltriphenylphosphonium chloride on the viability of cancer cell lines and normal cells. Cells were incubated 72 h in fresh medium (0) or with 0.1 µM of methyltriphenylphosphonium chloride (TPMP). Cell viability was measured spectrophotometrically in quadruplicates. Data are means ± SD (n=3).

Supplementary Figure 3

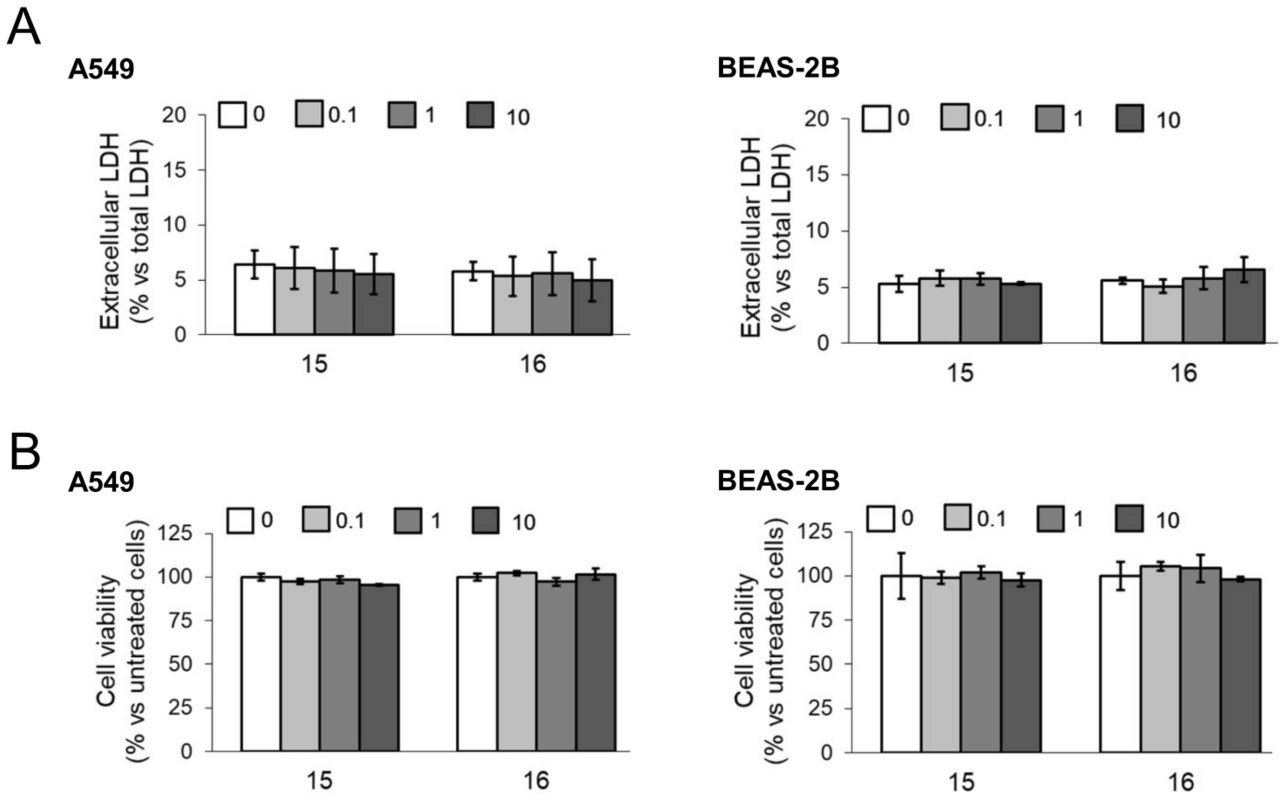

Supplementary Figure 3. Cytotoxic effects of compound **15** and **16**. A549 and BEAS-2B cells were grown in fresh medium (0) or incubated with 0.1, 1 or 10 µM of **15** and **16** for 24 h (panel **A**) or 72 h (panel **B**). **A.** The release of LDH was measured spectrophotometrically in duplicates. Data are means ± SD (n=3). **B.** Cell viability was measured spectrophotometrically in quadruplicates. Data are means ± SD (n=3).

Supplementary Figure 4

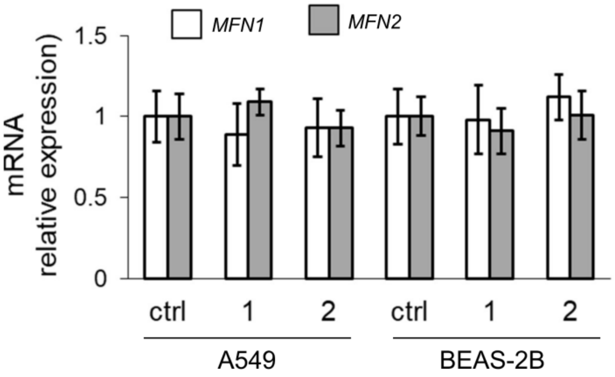

Supplementary Figure 4 Effects of TPP-derivatives on mitofusin 1 and mitofusin 2 expression. A549 and BEAS-2B cells were incubated 4 h in fresh medium (ctrl) or with 0.1 µM of **1** and **2**. Expression of mitofusin 1 (*MFN1*) and mitofusin 2 (*MFN2*) mRNA was evaluated by qRT-PCR in triplicates. Data are means ± SD (n=3).

Supplementary Figure 5

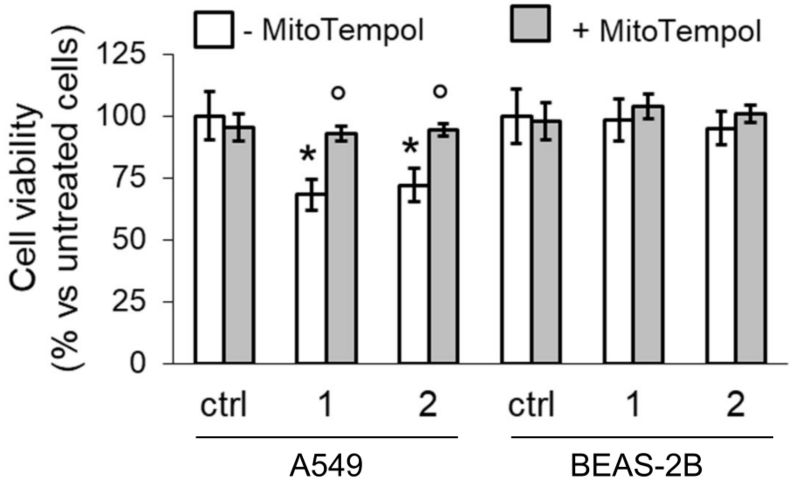

Supplementary Figure 5. Rescue of reduced viability induced by TPP-derivatives viability by MitoTempol. A549 and BEAS-2B cells were incubated 72 h in fresh medium (ctrl) or with 0.1 µM of **1** and **2**, in the absence (-) or presence (+) of 10 µM of the mitochondrial ROS scavenger MitoTempol. Cell viability was measured spectrophotometrically in quadruplicates. Data are means ± SD (n=3). * $p < 0.05$ *vs.* cells grown in fresh medium; ° $p < 0.05$: MitoTempol-treated cells *vs.* MitoTempol-untreated cells.
